# Supplementary material for: Improvement of image quality and assessment of respiratory motion for hepatocellular carcinoma with portal vein tumor thrombosis using contrast-enhanced four-dimensional dual-energy computed tomography
Source: PLoS One. 2021 Jan 22;16(1):e0244079. doi: 10.1371/journal.pone.0244079 (PMC7822294; doi:10.1371/journal.pone.0244079)
Supplement: Attachment — Submitted filename: Response to Reviewers.docx [file pone.0244079.s001.docx]

Dear Editor and Reviewers,

We would like to thank you for the insightful comments on our paper. We have revised the manuscript in the light of the comments of the editor and reviewers. The revisions have been made using the 'Edit' function of Word. We hope that the revised version of our manuscript is now suitable for publication in the *PLOS ONE*.

Point-by-point responses according to the reviewers’ comments are as follows:

Our response is in blue.

Editor’s Comments

Reviewers have now commented on your paper. You will see that they are advising that you revise in a minor way your manuscript.

If you are prepared to undertake the work required, I could reconsider my decision.

We have revised the manuscript in accordance with the reviewer’s comments. We hope that the revised version of our manuscript is now suitable for publication in the *PLOS ONE*.

Reviewer’s Comments

Please use the space provided to explain your answers to the questions above. You may also include additional comments for the author, including concerns about dual publication, research ethics, or publication ethics. (Please upload your review as an attachment if it exceeds 20,000 characters)

Reviewer #1: Overall, this research was well-designed. It suggested feasibility to used the CE-4D-DECT for better delineation of hepatocellular carcinoma with portal vein thrombosis. Authors have mentioned the limitation in phase of enhancement, which I agreed. Visibility of primary hepatocellular carcinoma and portal vein thrombosis is better in combined phases and the patterns of enhancement are various. Movement of tumor correlated with diaphragm is also point of concerned. Authors point out the discordance between diaphragm and tumor movement.

Thank you so much for your favorable comments. We have revised the manuscript in accordance with the reviewer’s comments. We hope that the revised version of our manuscript is now suitable for publication in the *PLOS ONE*.

I agreed but it would be better understand if authors would described more about how to measure tumor movement, eg.

The definition of GTV; Primary alone or combined with PVTT?

How to find the center of GTV? Was it contoured by radiation oncologist?

It would be appreciate if author have described it with figure, might be in supplement data.

We agree the reviewer’s comments, and the additional Figure was described to explain the tumor/diaphragm motion measurement. The tumor and diaphragm were delineated by radiation oncologists and a medical physicist, respectively. Radiation oncologists determined the GTV as the PVTT alone or PVTT combined with the primary tumor. The center of the tumor was calculated by using a treatment planning system (Eclipse, Varian Medical Systems). We believe that the readers can understand the methodology of the tumor/diaphragm motion in the revised manuscript. Thank you for your insightful comments.

In discussion, it would be outright if authors would add a paragraph mention about limitation of previously used or conventional technique in target delineation of hepatocellular carcinoma with some evidences.

We agree the reviewer’s comment. Although the CE-CT has been widely used for first-line diagnostic tests in patients suspected of having HCC, many major guidelines (such as European Association for the Study of the Liver and American Association for the Study of Liver Diseases) now include gadoxetic acid-enhanced MRI (GA-MRI) as a first-line diagnostic test. The limitation of conventional CE-CT is that conventional CT can only depict PTVV in the HU values. For the detection of PVTT using the GA-MRI, Bae et al. reported that the additional information of diffusion- and T2-weighted imaging could contribute better depiction of common imaging findings, such as continuity with the tumor in the adjacent liver parenchyma than CE-CT [Bae et al., Liver Cancer, 2020]. Previous study Kim et al demonstrated that the PVTT showed the increased T2 signal intensity and diffusion restriction, and the characteristic imaging features improved diagnostic capability [Kim et al., Radiology, 2016]. Moreover, the diffusion-weighted imaging has advantage for distinguishing bland thrombus from neoplastic thrombus in the portal vein in patients with HCC [Catalano et al., Radiology, 2010]. The evidences in detecting PVTT using MR imaging are added in the paragraph mentioned about limitation of previously used or conventional technique.
